# Subtypes of physical frailty and their long‐term outcomes: a longitudinal cohort study

**DOI:** 10.1002/jcsm.12577

**Published:** 2020-06-18

**Authors:** Shih‐Tsung Huang, Chikako Tange, Rei Otsuka, Yukiko Nishita, Li‐Ning Peng, Fei‐Yuan Hsiao, Makiko Tomida, Hiroshi Shimokata, Hidenori Arai, Liang‐Kung Chen

**Affiliations:** ^1^ Graduate Institute of Clinical Pharmacy National Taiwan University College of Medicine Taipei Taiwan; ^2^ Section of NILS‐LSA, National Center for Geriatrics and Gerontology Obu Japan; ^3^ Department of Epidemiology of Aging National Center for Geriatrics and Gerontology Obu Japan; ^4^ Department of Geriatrics, School of Medicine National Yang‐Ming University Taipei Taiwan; ^5^ Aging and Health Research Center National Yang‐Ming University Taipei Taiwan; ^6^ Center for Geriatrics and Gerontology Taipei Veterans General Hospital Taipei Taiwan; ^7^ School of Pharmacy National Taiwan University College of Medicine Taipei Taiwan; ^8^ Department of Pharmacy National Taiwan University Hospital Taipei Taiwan; ^9^ Graduate School of Nutritional Sciences Nagoya University of Arts and Sciences Nisshin Japan; ^10^ National Center for Geriatrics and Gerontology Obu Japan

**Keywords:** Aging, Subtypes of physical frailty, Mobility subtype frailty, Long‐term outcome, Group‐based multitrajectory model

## Abstract

**Background:**

Components of physical frailty cluster into subtypes, but it remains unknown how these might be associated with age‐related functional declines and multimorbidities. This study aims to investigated associations of physical frailty subtypes with functional declines and multimorbidity in a 10 year longitudinal cohort survey.

**Methods:**

Complementary longitudinal cohort study used group‐based multitrajectory modelling to verify whether frailty subtypes discovered in Taiwan are presented in another aging cohort, then investigated associations of these subtypes with cognitive decline and multimorbidity. Participants aged ≥50 years were recruited from the third to sixth waves (May 2002 to July 2010) of the National Institute for Longevity Sciences‐Longitudinal Study of Aging, in Japan. People with incomplete data, pre‐frail/frail status before their index wave, and those with incomplete data or who died during follow‐up, were excluded. Group‐based trajectory analysis denoted five established physical frailty criteria as time‐varying binary variables in each wave during follow‐up. Incident frailty was classified as mobility subtype (weakness/slowness), non‐mobility subtype (weight loss/exhaustion), or low physical activity subtype. General linear modelling investigated associations of these frailty subtypes with activities of daily living, digit symbol substitution test (DSST) and Charlson Comorbidity Index (CCI) at 2 year follow‐up.

**Results:**

We identified four longitudinal trajectories of physical frailty, which corroborated the distinct subtypes we discovered previously. Among 940 eligible participants, 38.0% were robust, 18.4% had mobility subtype frailty, 20.7% non‐mobility subtype, and 20.1% low physical activity subtype. People with mobility subtype frailty were older than those with other frailty subtypes or robust status and had higher prevalence of hypertension, diabetes, and heart failure. In the multivariable‐adjusted general linear models, mobility‐subtype frailty was associated with a significantly lower DSST score (point estimate −2.28, *P* = 0.03) and higher CCI (point estimate 0.82, *P* < 0.01) than the other groups.

**Conclusions:**

Mobility‐subtype frailty was associated with functional declines and progression of multimorbidity; the long‐term effects of physical frailty subtypes deserve further investigation.

## Introduction

Frailty is a prevalent geriatric syndrome, characterized by functional declines and depletion of physiologic reserves, which independently predicts adverse health outcomes and mortality in vulnerable older adults.^1^, ^2^ Due to its multifaceted aetiology, complex pathophysiology, and diverse clinical presentations, frailty has been defined operationally by various criteria and conceptual frameworks;^3^ current studies categorize frailty based on various domains of interest, such as physical, cognitive, and social frailty.^3^, ^4^, ^5^ Much research has focused on physical frailty, defined based on the phenotypic criteria of weakness, slowness, low physical activity, weight loss, and exhaustion.^6^ Although these five components may individually reflect different aetiologies, they also act in clusters, suggesting the hypothesis that common pathophysiological pathways underlie physical frailty.^7^ Latent class analysis of a longitudinal aging cohort in Taiwan identified three distinct subtypes of physical frailty: non‐mobility (weight loss and exhaustion), mobility (slowness and weakness), and low physical activity alone;^8^ clinical characteristics differed between frailty subtypes, and the mobility subtype was associated with significantly poorer clinical outcomes. These findings suggest that although frailty may be a common manifestation of advanced aging and dysregulated homeostasis, this may result from different pathoaetiologies. However, because the frailty status of individuals in a longitudinal study cohort may change during follow‐up, the results of subsequent latent class analyses may differ. Moreover, outcomes of interest should include declines in physical and cognitive function, and progression of multimorbidity, in addition to adverse clinical outcomes. Hence, we investigated associations of physical frailty subtypes with functional declines and multimorbidity in a 10 year longitudinal cohort survey.

## Methods

### Study data source and population sample

This retrospective cohort study excerpted data from the National Institute for Longevity Sciences‐Longitudinal Study of Aging (NILS‐LSA), a prospective longitudinal cohort study of community‐dwelling middle‐aged and elderly Japanese.^9^NILS‐LSA initially enrolled 2267 people 40–79 years old, stratified by age and sex, who were sampled at random from Obu City and Higashiura Town, near the National Center for Geriatrics and Gerontology, Aichi Prefecture, Japan. The NILS‐LSA first wave survey (November 1997 to April 2000) entailed detailed participant questionnaires and medical check‐ups, anthropometric measurements, physical fitness tests, and nutritional examinations at the NILS‐LSA Examination Center;^**9**^ biennial follow‐ups at the same institution continued until the seventh wave (July 2010 to July 2012). New participants aged 40–79 years, selected at random from the same residential areas, were recruited every year to provide age‐matched and sex‐matched replacements for participants (<80 years old) who were unable to attend the follow‐up surveys (e.g. due to moving elsewhere, dying, or for other reasons).^9^

**FIGURE 1:**
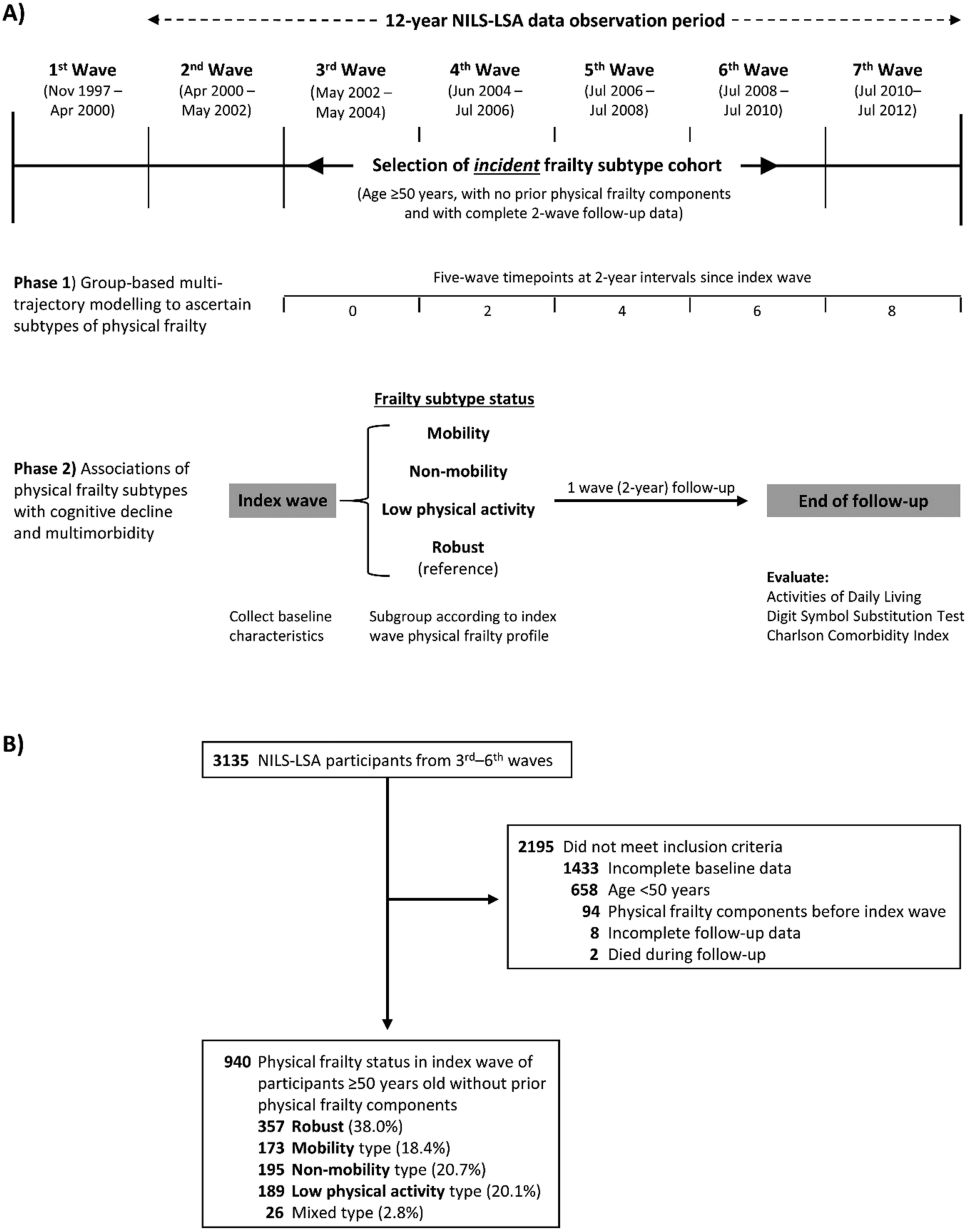
Study design and participant selection. (A) Scheme of study phases 1 and 2. (B) Selection of the incident frailty subtype cohort. NILS‐LSA, National Institute for longevity sciences‐longitudinal study of aging.

We analysed data acquired from NILS‐LSA participants aged ≥50 years during the second (April 2000 to May 2002) to sweventh waves, in two complementary studies (*Figure* 1); the analytic cohorts comprised eligible participants recruited in the third (May 2002 to May 2004) to the sixth (July 2008 to July 2010) waves. Study analyses excluded NILS‐LSA participants with incomplete data or any components of physical frailty in the wave before their index wave and those with incomplete data or who died during follow‐up.

## Ethical approval

The National Center for Geriatrics and Gerontology Committee on Ethics of Human Research approved the NILS‐LSA protocol. All NILS‐LSA participants gave written informed consent before any study‐related procedure ensued.

### Frailty phenotype and subtypes

Physical frailty was defined based on Cardiovascular Health Study (CHS) criteria, comprising exhaustion, weakness, slowness, low physical activity, and weight loss,^6^, ^8^, ^10^ which were modified consistent with the inceptive physical frailty subtype study.^8^ Exhaustion was defined based on responses to two questions from the Center for Epidemiologic Studies Depression Scale (CES‐D) questionnaire:^11^, ^12^ ‘Q7: I felt that everything I did was an effort’, and ‘Q20: I could not get “going”’. Possible responses included ‘1. Rarely or none of the time (less than 1 day)’, ‘2. Some or a little of the time (1–2 days)’, ‘3. Occasionally or a moderate amount of time (3–4 days)’ or ‘4. Most or all of the time (5–7 days)’; due to the issue of Japanese language translation, only participants who answered 3 or 4 to Q7, or 2, 3, or 4 to Q20, were deemed to meet the criterion of exhaustion. Weakness, slowness, and low physical activity were defined as the lowest quintile of all participants by sex, which was equivalent to the original CHS criterion,^6^ in terms of handgrip strength (kg), walking speed (m/s), and leisure‐time physical activity level, using a questionnaire modified for use in Japan from the Minnesota Leisure‐time Physical Activity Questionnaire.^13^ Because NILS‐LSA examinations were biennial, weight loss was defined as unintentionally losing >5 kg over the prior 2 years.

Physical frailty subtypes were defined based on the inceptive study:^8^ individuals with weakness or slowness but not weight loss or exhaustion were classified as having the mobility frailty subtype; those with weight loss or exhaustion but not weakness or slowness were classified as having non‐mobility frailty subtype; and low physical activity only constituted the third frailty subtype. Participants not meeting any CHS frailty criteria were classified as robust.

### Phase 1: group‐based multitrajectory modelling to ascertain physical frailty subtypes

In the first phase, assuming that physical frailty status may change over time, we used group‐based multiple trajectory modelling^14^ with NILS‐LSA data as external validation to characterize patterns of physical frailty and identify groups of subjects with distinctive phenotypic presentations over time, specifically to ascertain whether three physical frailty subtypes discovered in our previous study (i.e. mobility, non‐mobility, and low physical activity) were also present in the NILS‐LSA aging cohort in Japan. The index wave from which subjects' baseline data were acquired was defined as the start of follow‐up, which ended on completing the seventh wave, and all five components of physical frailty were denoted as time‐varying binary variables in each wave during follow‐up (*Figure* 1A).

### Phase 2: associations of physical frailty subtypes with cognitive decline and multimorbidity

In the second phase, having validated that the mobility, non‐mobility and low physical activity frailty subtypes were also present in Japan, and we identified subjects with incidence of these three physical frailty subtypes between the third to sixth waves, and compared them with robust people without any CHS criteria to investigate associations between incident physical frailty and declines in physical and cognitive function, and progression of multimorbidity. People with multiple physical frailty components who could not be categorized into any of these frailty subtype groups were classified as mixed subtype and excluded from subsequent analyses. Follow‐up for each subject started at their index wave and ended on completing the next wave, yielding one‐wave (2 years) follow‐up period for all subjects (*Figure* 1B).

### Outcome measurements

Outcomes of interest in the study of associations between frailty subtypes and physical and cognitive decline included activities of daily living (ADL), digit symbol substitution test (DSST) performance, and the modified Charlson Comorbidity Index (CCI), which is a validated weighted multimorbidity score derived from self‐reported medical history.^15^, ^16^ ADL was scored according to the Tokyo Metropolitan Institute of Gerontology Index of Competence, which comprises 13 measures of physical and social function.^17^, ^18^ DSST is one of four subtests in the Wechsler Adult Intelligence Scale‐Revised Short Form,^19^, ^20^ which estimates overall levels of general intellectual functioning; previous studies have shown that the DSST score is associated with memory and social recognition, especially in executive function and working memory.^21^

### Other variables

Demographic data included subjects' age, sex, smoking status, alcohol consumption, education level, and income. Height and weight, measured using digital scales with participants lightly clothed, were used to calculate body mass index (kg/m^2^). Health status and multimorbidity (hypertension, diabetes, hyperlipidaemia, stroke, ischemic heart disease, heart failure, liver disease, kidney disease, gastric or duodenal ulcer, chronic bronchitis, rheumatoid arthritis, cancer, dementia, gout or hyperuricemia, and osteoporosis) were assessed based on self‐completed questionnaires and confirmed by subsequent medical examinations. Trained interviewers used a questionnaire to assess the Mini‐Mental State Examination score and the CES‐D score.

### Statistical analysis

All analyses were performed using sas, Version 9.3 (SAS Institute Inc., Cary, NC). The first study phase classified trajectories of physical frailty subtypes using the group‐based multitrajectory model,^14^ which uses finite‐mixture modelling to reveal latent clusters of individuals following similar trajectories across multiple indicators over time. The statistical macro, ‘PROC TRAJ’,^22^ which is a free add‐on to SAS, was used to fit the group‐based multitrajectory model of frailty components during longitudinal follow‐up and classify subjects into distinct physical frailty subtypes (mobility type, non‐mobility type, and low physical activity type). We used the Bayesian information criterion (BIC) value as a selection index to compare different models with varying numbers of groups and trajectory shapes; each trajectory group was required to include >5% of participants, and the model with the highest BIC value was considered the best.

Categorical variables were compared by Chi‐square tests and continuous variables by analysis of variance (ANOVA); however, Fisher exact test was used to compare categorical variables if any data value in 2 × 2 Chi‐square tables was <5. General linear models were used to examine associations of physical frailty subtypes with ADL, DSST, and CCI, with multivariable adjustment; associations were presented as point estimates, with *P* values (α = 0.05).

## Results

**FIGURE 2:**
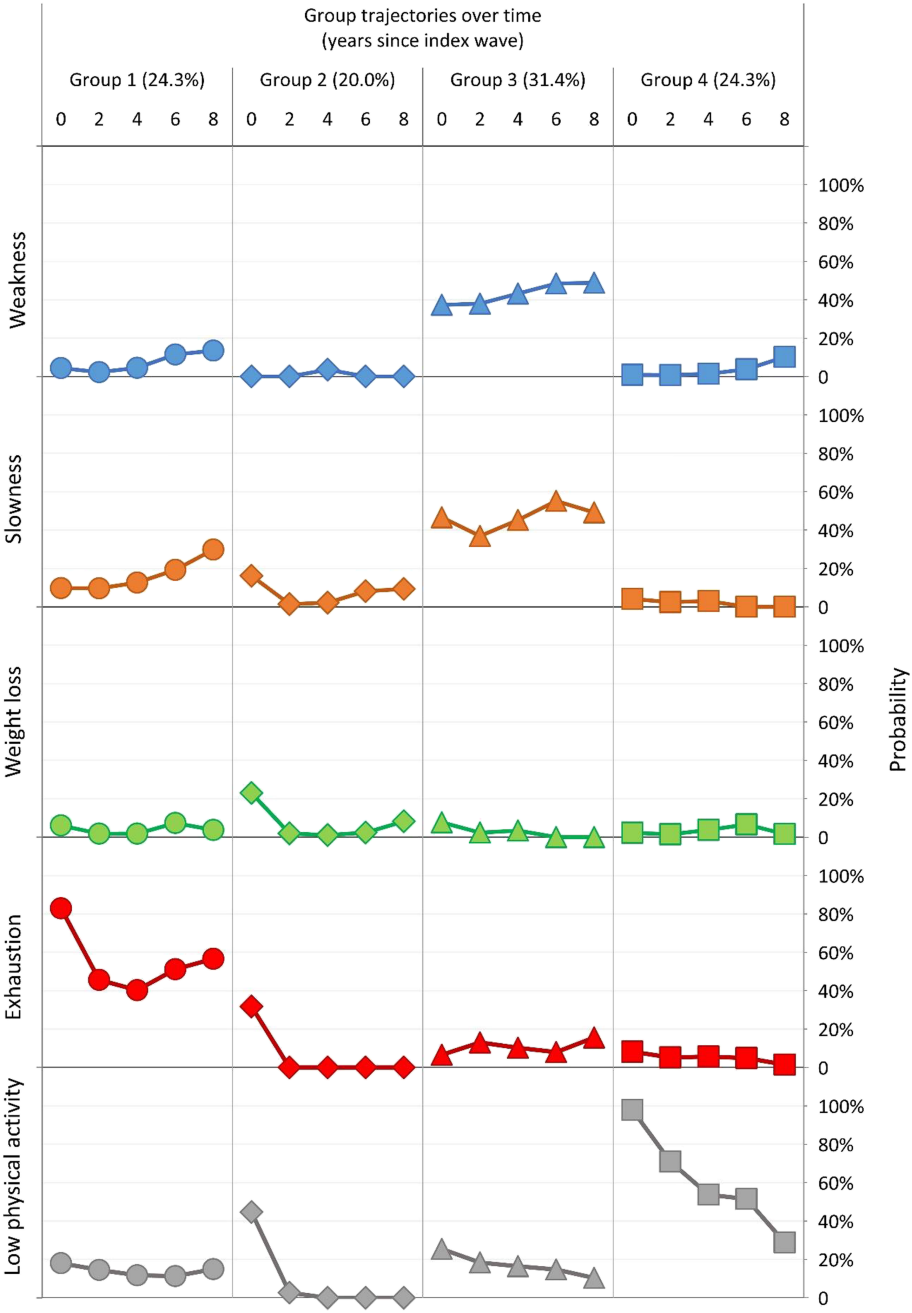
Results of group‐based multitrajectory modelling: estimated incident probabilities of five physical frailty component.

Group‐based multitrajectory modelling in phase 1 involved 728 eligible subjects (mean age 63.6 years, mean follow‐up 3.5 waves) selected from among 3135 NILS‐LSA third to sixth wave participants. Although group‐based multitrajectory model analysis and selection identified a model with five physical frailty trajectory patterns over longitudinal follow‐up as being the best based on BIC values (five‐trajectory model: BIC = −3263.6; four‐trajectory model: BIC = −3342.3; and three‐trajectory model: BIC = −3317.1), it was too complex to be clinically meaningful and yielded a trajectory with only 5% of participants; therefore, based on clinical expert opinion, the four‐trajectory model was chosen for the final model (*Figure* 2). Three of these four trajectories appeared similar to the mobility, non‐mobility, and low physical activity subtypes operationally classified in our previous study.^8^ Group 1 (24.3%) comprised subjects distinguished by higher probability of exhaustion over time; Group 3 (31.4%) had relatively high probability of incident weakness and slowness; Group 4 (24.3%) had only high probability of physical activity diminution. As inter‐class shifts between different subtypes of physical frailty were infrequent, these subtypes remained relatively stable over time.

In phase 2 study, 940/3135NILS‐LSA third to sixth wave participants who met the inclusion criteria were classified into mobility frailty subtype (*n* = 173, 18.4%), non‐mobility frailty subtype (*n* = 195, 20.7%), low physical activity frailty subtype, mixed subtype (*n* = 26, 2.8%), and robust (*n* = 357, 38.0%) based on incident physical frailty in the index wave (*Figure* 1B). Participants who classified as mixed type were not included in subsequent analysis. The baseline characteristics of participants with each subtype of physical frailty differed significantly between groups (*Table* 1); people with the mobility frailty subtype were older and had higher CCI scores and lower Mini‐Mental State Examination score than those with other subtypes or who were robust (*P* < 0.01), with the highest prevalence rates of hypertension (*P* < 0.01), diabetes (*P* < 0.01), and ischemic heart disease (*P* = 0.03). People with the non‐mobility frailty subtype had the highest CES‐D score (*P* < 0.01) and prevalence of hyperlipidaemia (*P* = 0.05).

**TABLE 1:** Baseline characteristics of the incident frailty subtype group

Data values show *M* ± *SD*, or number (%)		Physical frailty subtype classification				*P* value
		Robust	Mobility	Non‐mobility	Low physical activity	
Number		357	173	195	189	
Age (years)		61.4 ± 7.2	68.7 ± 8.2	63.2 ± 7.4	61.2 ± 6.9	<0.01
Sex (Female)		152 (42.6)	77 (44.5)	100 (51.3)	94 (49.7)	0.17
Height (cm)		161.0 ± 8.0	157.2 ± 8.8	159.4 ± 8.5	159.9 ± 8.1	<0.01
Weight (kg)		59.4 ± 9.7	56.7 ± 9.0	58.3 ± 9.8	59.5 ± 9.7	0.01
Body mass index (kg/m^2^)		22.8 ± 2.6	22.9 ± 2.8	22.9 ± 2.8	23.2 ± 2.8	0.43
Body fat (%)		24.9 ± 6.6	27.1 ± 6.7	26.5 ± 6.8	27.2 ± 6.4	<0.01
Bone mineral density (g/cm^2^)		1.1 ± 0.1	1.0 ± 0.1	1.0 ± 0.1	1.0 ± 0.1	<0.01
MMSE score		28.3 ± 1.7	27.7 ± 1.8	28.4 ± 1.6	28.2 ± 1.5	<0.01
CES‐D Scale		4.0 ± 3.9	5.3 ± 4.7	12.5 ± 7.7	5.2 ± 4.8	<0.01
Alcohol intake (g/day)		12.1 ± 18.4	10.3 ± 16.1	9.5 ± 14.8	10.6 ± 18.0	0.38
Smoking status	Never	200 (56.0)	101 (58.4)	112 (57.4)	109 (57.7)	0.79
	Previous	111 (31.1)	47 (27.2)	56 (28.7)	48 (25.4)	
	Current	46 (12.9)	25 (14.4)	27 (13.9)	32 (16.9)	
Education (years)		12.4 ± 2.7	11.6 ± 2.7	12.4 ± 2.7	12.3 ± 2.7	0.01
Income (million Yen)	<1.5	4 (1.1)	9 (5.2)	5 (2.6)	3 (1.6)	<0.01
	1.5–4.5	90 (25.2)	76 (43.9)	65 (33.3)	47 (24.9)	
	>4.5	262 (73.4)	84 (48.6)	119 (61.0)	137 (72.5)	
Occupation	None	72 (20.2)	73 (42.2)	46 (23.6)	24 (12.7)	<0.01
	Homemaker	69 (19.3)	34 (19.7)	43 (22.1)	28 (14.8)	
	Employed	216 (60.5)	66 (38.2)	106 (54.4)	137 (72.5)	
Health status (self‐reported)	Good	172 (48.2)	58 (33.7)	48 (24.6)	53 (28.0)	<0.01
	Normal	179 (50.1)	109 (63.4)	127 (65.1)	122 (64.6)	
	Bad	6 (1.7)	5 (2.9)	20 (10.3)	14 (7.4)	
Charlson Comorbidity Index		1.1 ± 1.7	1.9 ± 2.2	1.2 ± 1.7	1.1 ± 1.8	<0.01
Hypertension		97 (27.2)	72 (41.6)	56 (28.7)	51 (27.0)	<0.01
Diabetes		17 (4.8)	26 (15.0)	14 (7.2)	12 (6.4)	<0.01
Dyslipidaemia		72 (20.2)	37 (21.4)	56 (28.7)	34 (18.0)	0.05
Stroke		8 (2.2)	9 (5.2)	6 (3.1)	2 (1.1)	0.10
Ischemic heart disease		12 (3.4)	15 (8.7)	11 (5.6)	6 (3.2)	0.03
Heart failure		46 (12.9)	35 (20.2)	30 (15.4)	28 (14.8)	0.18
Liver disease		17 (4.8)	12 (6.9)	11 (5.6)	9 (4.8)	0.74
Kidney disease		19 (5.3)	4 (2.3)	10 (5.1)	8 (4.2)	0.44
Gastric/duodenal ulcer		58 (16.3)	24 (13.9)	34 (17.4)	21 (11.1)	0.30
Chronic bronchitis		3 (0.84)	4 (2.3)	1 (0.51)	3 (1.6)	0.43
Rheumatoid arthritis		17 (4.8)	13 (7.5)	5 (2.6)	8 (4.2)	0.16
Cancer		11 (3.1)	15 (8.7)	4 (2.1)	7 (3.7)	0.01
Dementia		1 (0.3)	0.0 (0)	0 (0.0)	0 (0.0)	1.00
Osteoporosis		13 (3.6)	12 (6.9)	10 (5.1)	14 (7.4)	0.21
Gout or hyperuricemia		16 (4.5)	7 (4.1)	15 (7.7)	10 (5.3)	0.35

CES‐D, Center for Epidemiologic Studies of Depression; MMSE, Mini‐Mental State Examination; SD, standard deviation.

In the multivariable‐adjusted general linear models, people with the mobility frailty subtype had significantly lower DSST scores (point estimate = −2.24, *P* = 0.04) and higher CCI scores (point estimate = 0.83, *P* < 0.01) than robust people (*Table* 2)

**TABLE 2:** Association of frailty subtypes with functional and cognitive performance, and comorbidity

Number	Physical frailty subtype classification						
	Robust	Mobility		Non‐mobility		Low physical activity	
	357	173		195		189	
		Point estimate	*P* value	Point estimate	*P* value	Point estimate	*P* value
Activities of daily living^a^	Reference	−0.13	0.25	0.05	0.66	−0.09	0.38
Digit symbol substitution test^a^	Reference	−2.24	0.04	1.50	0.18	−1.21	0.22
Charlson Comorbidity Index^b^	Reference	0.83	<0.01	0.09	0.64	0.23	0.19

^a^ Adjusted by age, sex, Center for Epidemiologic Studies Depression Scale, and Charlson Comorbidity Index in the index wave.

^b^ Adjusted by age, sex, and Center for Epidemiologic Studies Depression Scale.

## Discussion

This study clearly corroborated the existence of distinct subtypes of physical frailty, with characteristics and clinical outcomes among community‐dwelling Japanese people older than 50 that were very similar to those reported previously by a longitudinal aging cohort study in Taiwan.^8^

The inceptive latent class analysis suggested that physical frailty can be subclassified as mobility type, non‐mobility type, and physical inactivity type.^8^ Although latent class analysis clearly differentiated subjects based on clusters of physical frailty components, categorization based on extant frailty components made it difficult to conclude that such clusters developed naturally over time. However, as all participants in our study were free from any physical frailty component when enrolled, we could affirm that these frailty subtypes indeed developed over time, which may imply specific pathophysiologic processes associated with aging. Moreover, longitudinal follow‐up supported the hypothesis that each physical frailty subtype, especially the mobility subtype, would have different clinical outcomes.

The pathoaetiology of physical frailty involves comixed biological, psychological, clinical, social, and other factors.^3^, ^4^, ^5^, ^23^, ^24^, ^25^, ^26^, ^27^ Inflammation has long been considered a principal underlying cause,^28^ and numerous inflammatory cytokines have been associated with physical frailty, notably interleukin‐6, tumour necrosis factor‐alpha, and soluble intercellular adhesion molecule‐1.^29^ Our latest findings, however, suggest that ‘inflammaging’ is only part of the story; based on comparisons of demographic characteristics and long‐term outcomes, we contend that mobility‐type frailty may have neuro‐musculoskeletal causality, while non‐mobility type frailty may be associated with not only inflammation but also depressed mood and malnutrition.^29^, ^30^ Moreover, subtypes of physical frailty, especially the mobility subtype, were associated with cognitive performance and multimorbidity. DSST performance, which is associated with executive function and working memory,^21^ may decline subsequent to developing mobility‐type frailty. This distinct phenotype is consistent with the concept of ‘cognitive frailty’ proposed in earlier studies.^31^, ^32^Age‐related declines in physical function (i.e.mobility‐subtype frailty) may precede cognitive impairments in executive function and working memory.^33^ This evidence is consistent with previous findings and highlights the reciprocity of age‐related physical and cognitive declines that define cognitive frailty, which clearly increases mortality risk.^32^, ^34^ We therefore propose that cognitive frailty may be a consequence of mobility‐subtype physical frailty that results from a specific pathoaetiologic aging pathway.

Physical frailty and cognitive impairment often coexist and may progressively worsen with advancing age.^35^, ^36^, ^37^, ^38^, ^39^ In longitudinal studies, impaired executive function and working memory were the first signs of cognitive decline presaging Alzheimer's disease, appearing earlier than short‐term memory deficits.^40^, ^41^ The predementia syndrome, motoric cognitive risk syndrome, which is characterized by slow gait and cognitive impairments,^42^ might be a specific phenotype of the mobility frailty subtype that manifests later on. We have advocated using objective versus subjective measurements of cognitive impairment to define cognitive frailty, and it seems expedient to identify non‐memory deficits, particularly in executive function, language, and working memory.^36^ Synthesizing available evidence, we postulate that the physical frailty mobility subtype is the earliest manifestation of this specific degenerative pathology, and that it is followed by cognitive frailty and motoric cognitive risk syndrome.

The results of this study should be interpreted in light of some limitations. First, the NILS‐LSA cohort study is focused on healthy aging and participants withdrew if their perceived health status declined. Therefore, the mortality rate and ADL declines during follow‐up were trivial, which limited investigation of some endpoints. Nevertheless, early declines in cognitive function and progression of multimorbidity still reached statistical significance. Although we only measured outcomes 2 years after the index wave, we still found that people with mobility‐subtype physical frailty had significantly lower DSST scores and higher CCI compared with robust counterparts at follow‐up. Third, our study population is relatively young with 8.53% of them were older than 75 years old. Some associations, such as the mobility subtypes and incident ADL, were not observed in this study due to the relatively young age of our study subjects. Nevertheless, as we hypothesized that frailty subtype could be a precursor of disability, cognitive impairment, or poor health status, early identification of people with these frailty subtypes in younger age may provide clinicians with opportunities for timely interventions.

## Conclusions

This study provided new evidence corroborating the existence of distinct physical frailty subtypes associated with aging and further demonstrated that these subtypes were phenotypically stable over time. Mobility‐subtype frailty was significantly associated with functional declines and progression of multimorbidity. These findings warrant further research to evaluate the long‐term outcomes of physical frailty subtypes and associated functional declines.

## Conflict of interest

S.T.H., F.Y.H., and L.K.C. have received research grants from Ministry of Science and Technology (MOST 107–2634‐F‐010‐001), Taiwan. C.T., R.O., Y.N., L.N.P., M.T., H.S., and H.A. declare that they have no conflict of interest.

## Funding

This research was supported by the Ministry of Science and Technology, Executive Yuan of Taiwan (MOST 107‐2634‐F‐010‐001).

## Contributors

All the authors designed the research, drafted the article, revised it critically for important intellectual content, and approved the final version to be published. Huang ST and Chen LK wrote the paper. Huang ST performed the literature search and analysed data. Tange C, Arai H, and Chen LK provided critical methodological inputs. Tange C, Otsuka R, Nishita Y, Peng LN, Hsiao FY, Tomida M and Hiroshi S provided methodological and statistical inputs. Chen LK and Arai H contributed to the clinical interpretation. Chen LK is guarantor.
